# GTPase GPN3 facilitates cell proliferation and migration in non-small cell lung cancer by impeding clathrin-mediated endocytosis of EGFR

**DOI:** 10.1038/s41420-025-02317-y

**Published:** 2025-02-01

**Authors:** Linlin Xu, Jiankun Guo, Xinsheng Xie, Hailong Wang, Alan Jiang, Changhua Huang, Hua Yang, Shiwen Luo, Limin Chen

**Affiliations:** 1 Medical Innovation Center, The First Affiliated Hospital, Jiangxi Medical College, Nanchang University, Nanchang, 330006 Jiangxi China; 2 Department of Pathology, The First Affiliated Hospital, Jiangxi Medical College, Nanchang University, Nanchang, 330006 Jiangxi China; 3 Center for Experimental Medicine, The First Affiliated Hospital, Jiangxi Medical College, Nanchang University, Nanchang, 330006 Jiangxi China

**Keywords:** Endocytosis, Non-small-cell lung cancer

## Abstract

Small GTPases play a critical role as regulatory molecules in signaling transduction and various cellular processes, contributing to the development of human diseases, including cancers. GPN3, an evolutionarily conserved member of the GPN-loop GTPase subfamily classified in 2007 according to its structure, has limited knowledge regarding its cellular functions and molecular mechanisms. In this study, we demonstrate that GPN3 interacts with clathrin light chain A (CLTA), a vesicle coat protein, as well as clathrin-mediated endocytosis associated modulators AP2B1 and AP2S1. Upregulation of GPN3 leads to the inhibition of clathrin-coated pit invagination. Furthermore, we discovered that GPN3 interacts with the epidermal growth factor receptor (EGFR) and regulates the co-localization of EGFR and CLTA, as well as the localization of EGFR in early endosomes upon EGF stimulation. As a result, this leads to a decrease in endocytic levels of EGFR and an increase in the accumulation of EGFR on the cell membrane surface, thereby prolonging activation of EGFR signaling. The functional effects exerted by GPN3 are dependent on cellular levels of GTP abundance. Furthermore, our findings indicate that aberrant overexpression of GPN3 is observed in non-small cell lung cancer (NSCLC) tissues compared to adjacent normal tissues, and high expression levels of GPN3 are associated with poor prognosis for NSCLC patients. Collectively, these findings reveal that GPN3 acts as an oncogene promoting cell proliferation and migration in NSCLC through regulation of clathrin-dependent EGFR endocytosis. These results suggest that targeting GPN3 could serve as a novel prognostic biomarker and therapeutic strategy for NSCLC treatment.

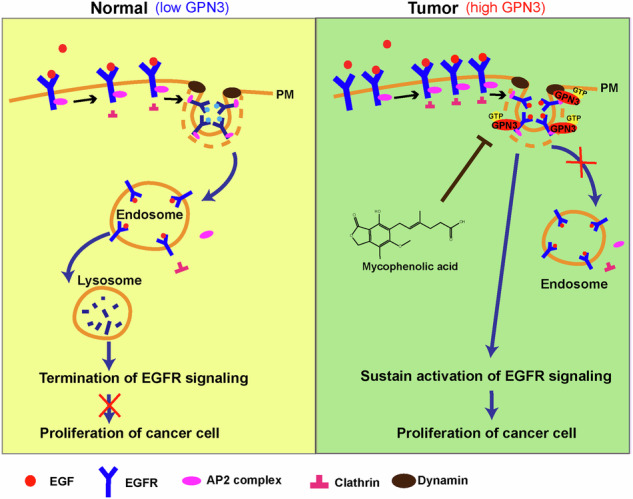

## Introduction

Lung cancer remains one of the most frequently diagnosed malignancies and is highly lethal [1]. Non-small cell lung cancer (NSCLC) accounts for 85% of lung cancer cases, comprising two main subtypes, namely lung adenocarcinoma (LUAD) and lung squamous carcinoma (LUSC) [2]. Despite available treatment options such as surgery, chemotherapy, irradiation, immunotherapy, and targeted therapy, patient survival rates remain unsatisfactory due to early metastases, acquired chemoresistance and radioresistance, as well as immunogenic heterogeneity [3]. Therefore, it is crucial to further explore the molecular mechanisms underlying NSCLC progression in order to develop novel therapeutic strategies that can improve patient outcomes.

Small GTPases are critical signaling regulatory molecules involved in various cellular processes through switching between a GTP-bound active conformation and a GDP-bound inactive conformation [4, 5]. These functions have been extensively studied in translation regulation, signaling transduction pathways, intracellular transport mechanisms, protein localization processes, membrane transport systems etc. [4, 6, 7]. In addition to the well-known Ras superfamily of phosphate-binding loop (P-loop) GTPases [6], there exists another subfamily called GPN-loop GTPases which were defined in 2007 [8]. However, little is known about the cellular functions and molecular mechanisms associated with this particular subfamily of GTPases. One member of this subfamily, namely GPN3, has been found to be evolutionarily highly conserved across species and plays an essential role in RNA polymerase II biogenesis [9, 10]. Furthermore, GPN3 interacts with RNA polymerase II facilitating its nuclear import [11, 12], suggesting that it may also mediate protein nucleocytoplasmic transport processes. Nevertheless, the precise cellular roles mediated by GPN3 still require further investigation. To date, only two studies have reported an association between GPN3 and cancer progression, demonstrating its role in regulating drug resistance in small cell lung cancer cells [13] and promoting breast cancer cell proliferation [14]. Therefore, further investigation is required to elucidate the roles and molecular mechanisms of GPN3 in tumors, including its potential involvement in NSCLC progression.

Aberrant expression and abnormal intracellular trafficking of epidermal growth factor receptor (EGFR) are well-established factors contributing to tumorigenesis, particularly in NSCLC [15]. Receptor tyrosine kinases, such as EGFR, typically undergo clathrin-dependent endocytosis through clathrin-coated pits (CCPs) and vesicles before being transported to early endosomes via flat clathrin microdomains [16–18]. While EGFR signaling is primarily activated at the plasma membrane, endocytosis serves as a negative feedback regulatory mechanism [18, 19]. Dysregulated endocytic trafficking of EGFR can result in constitutively active EGFR signaling implicated in cancer pathogenesis [20, 21]. As mentioned earlier, GTPases play a crucial role in membrane trafficking and protein localization [22] while GPN3 mediates nucleocytoplasmic transport of RNA polymerase II [12]. Henceforth, we hypothesize that GPN3 may regulate endocytosis-mediated protein trafficking warranting further exploration.

In this study, our findings demonstrate that GPN3 inhibits the endocytic transport of EGFR by modulating CCPs invagination thereby prolonging activation of EGFR signaling pathway. Additionally, GPN3 promotes NSCLC cell proliferation and migration, and we observe aberrant overexpression of GPN3 in NSCLC tissues compared with paired adjacent normal tissues, high levels of GPN3 are associated with poor prognosis among NSCLC patients. Moreover, the functions of GPN3 are dependent on the cellular GTP abundance because depletion of GTP could inhibit the interaction between GPN3 and EGFR and reverse the activation of EGFR signaling mediated by GPN3. Therefore, GPN3 may serve as a potential prognostic biomarker and therapeutic target for NSCLC treatment.

## Results

### GPN3 regulates clathrin-mediated endocytosis

**Fig. 1:**
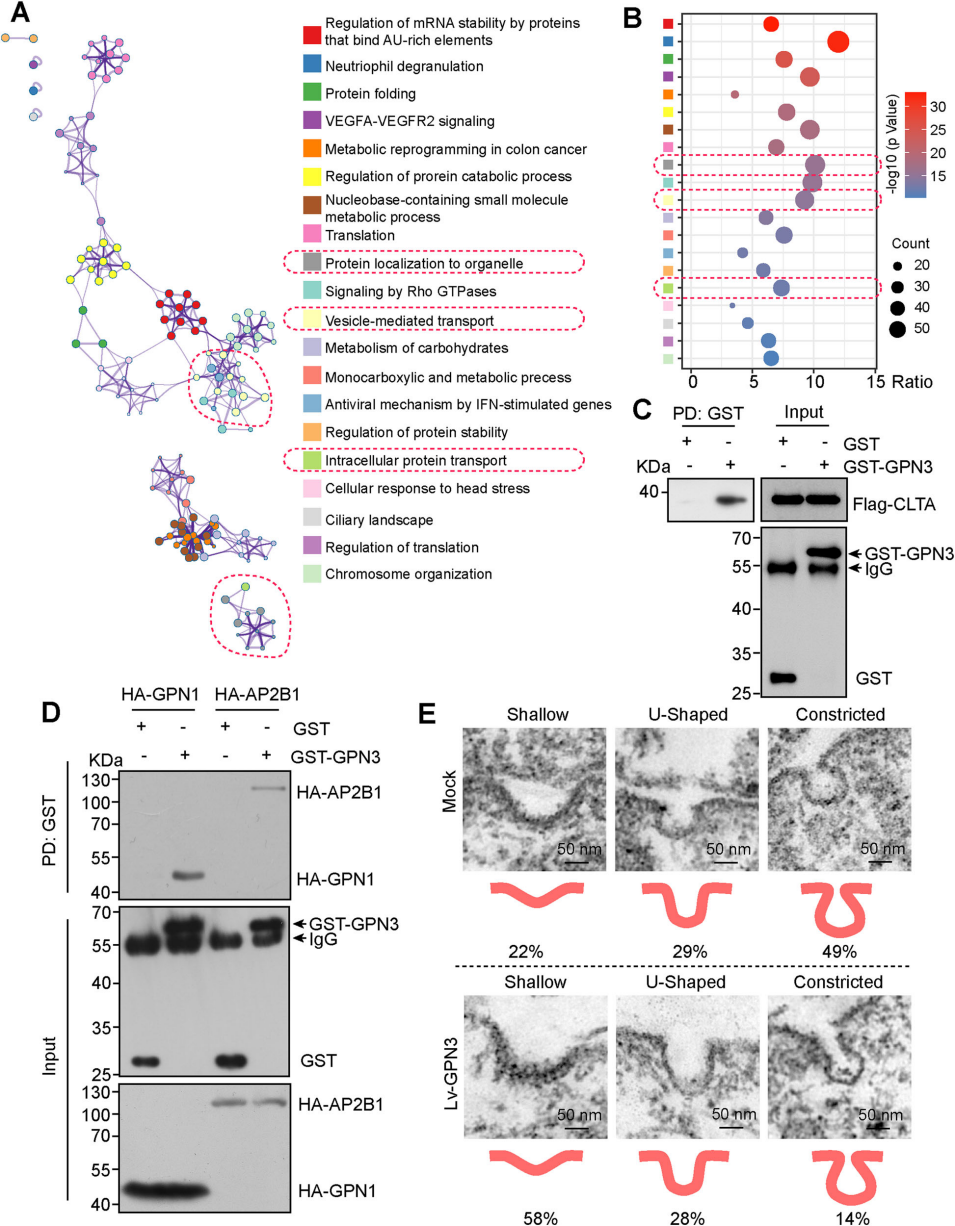
GPN3 regulates clathrin-mediated endocytosis. **A** Pathway and process enrichment analysis of GPN3 potential interacted hits identified by LC-MS/MS assay. 293T cells transfected with GPN3-overexpressing plasmid or control vector were lysed and used to Co-IP assay and followed LC-MS/MS assay. The identified potential interacted hits of GPN3 were subjected to enrichment analysis using Metascape software. **B** Bubble plot illustrating the pathways and processes mentioned in (**A**). **C** GST-pull down assay demonstrated the interaction between GPN3 and CLTA. **D** GST-pull down assay confirmed the interaction between GPN3 and AP2B1, with GPN1 serving as a positive control. **E** NCI-H1299 cells stably overexpressing GPN3 and the control cells were prepared for resin-embedded thin section, and CCPs were randomly traced and captured under a transmission electron microscope. 30–40 CCPs were quantified for each group.

To investigate the molecular mechanisms underlying GPN3 regulation, we identified the immunoprecipitate of GPN3 and a negative control (Flag) using LC-MS/MS analysis. Hits that were exclusively identified in the GPN3 group or had an abundance more than three times higher than that in the Flag group (475 hits) underwent pathway and process enrichment analysis through Metascape software [23]. The top 20 enriched terms (clusters) were shown in Figs. 1A, B and S1A, the identified hits were involved in translation, regulation of translation, mRNA stability regulation, and protein localization to organelle etc., consistent with previously reported roles of GPN3 in RNA polymerase II biogenesis and nuclear import [10, 12], indicating reliability of our data. Intriguingly, we also observed significant involvement of these hits in vesicle-mediated transport and intracellular protein transport (Fig. 1A, B). Furthermore, they participated in signaling pathways mediated by Rho GTPase (Fig. 1A, B). Rho GTPase, a subfamily of Ras superfamily, has well-known roles in vesicle trafficking [7, 22]. Hence, we hypothesized that GPN3 may be involved in membrane trafficking and protein localization.

Considering the vesicle coated proteins associated with clathrin complex, we investigated whether GPN3 regulates clathrin-mediated endocytosis. Firstly, we confirmed that GPN3 interacted with CLTA (clathrin light chain A) via GST-pull down assay (Fig. 1C). And, GPN3 also interacted with AP2B1 (AP-2 complex subunit beta) and AP2S1 (AP-2 complex subunit sigma), GPN1 was used as a positive interacting partner of GPN3 (Figs. 1D and S1B). AP2B1 and AP2S1 are components of adaptor complex 2 (AP2) involved in the formation of CCPs regulating clathrin-mediated endocytosis [24]. Intriguingly, upregulation of GPN3 inhibited the dynamic process of CCPs formation under EGF ligand stimulation (Fig. 1E). Therefore, our findings suggest that GPN3 may play a role in regulating signaling transduction of membrane receptors by modulating clathrin-mediated endocytosis.

### Upregulation of GPN3 inhibits EGF-induced EGFR endocytic trafficking

**Fig. 2:**
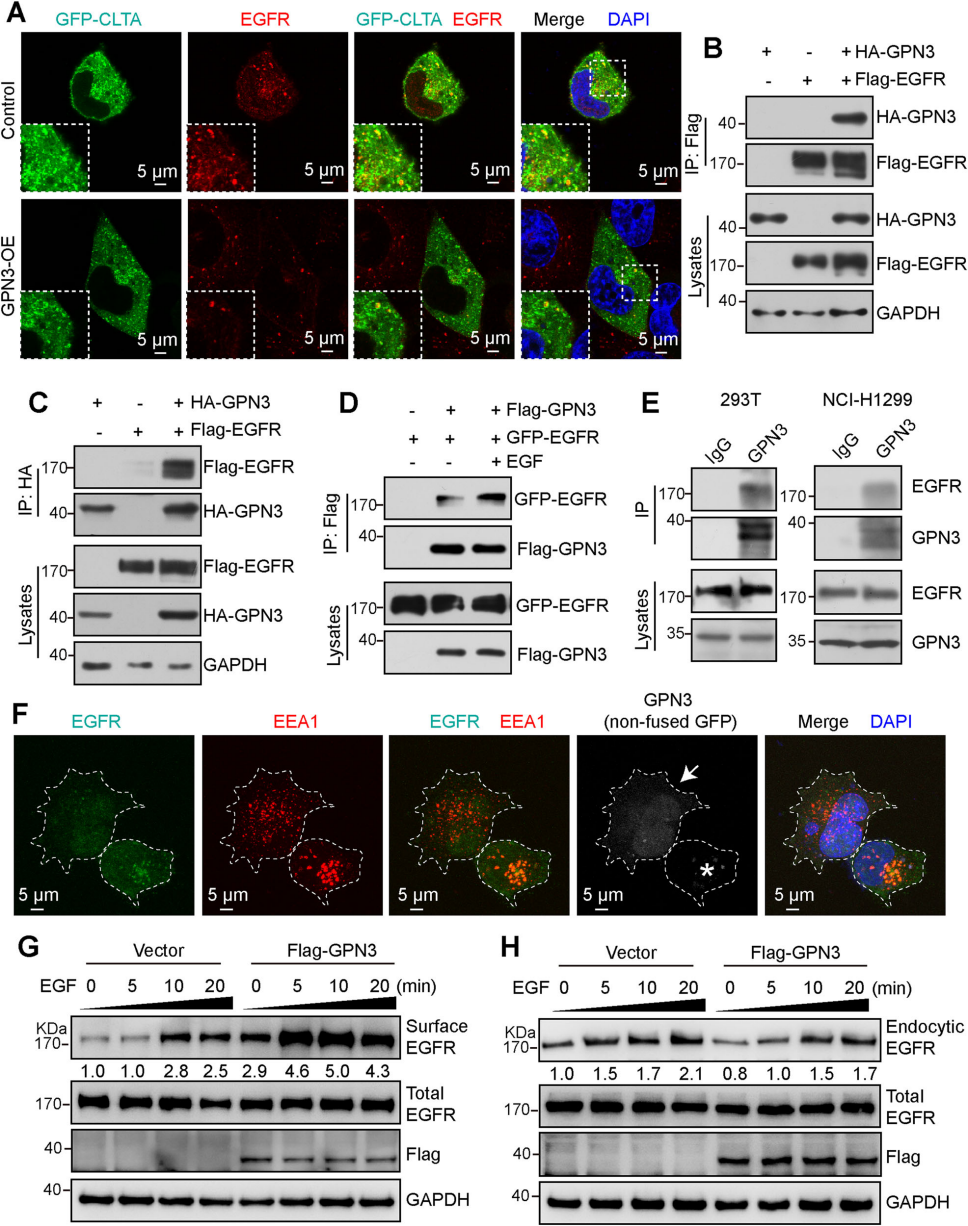
GPN3 upregulation inhibits EGF-induced EGFR endocytic trafficking. **A** Overexpression of GPN3 attenuated the colocalization between EGFR and CLTA. NCI-H1299 cells stably overexpressing GPN3 and control cells were transfected with GFP-CLTA expressing plasmid, followed by EGFR immunofluorescent staining. The dashed box indicates the enlarged inset. **B**, **C** GPN3 interacted with EGFR. 293T cells transfected with HA-GPN3, Flag-EGFR, or control vector were lysed and subjected to immunoprecipitation using anti-Flag (**B**) or anti-HA (**C**) magnetic bead. **D** The interaction between GPN3 and EGFR increased upon EGF stimulation. 293T cells transfected with Flag-GPN3, GFP-EGFR, or control vector were treated with 10 ng/ml EGF for 5 min before performing Co-IP assay. **E** Endogenous GPN3 interacts with EGFR both in 293T cells and NCI-H1299 cells. **F** Upregulation of GPN3 inhibited the localization of EGFR in early endosome. NCI-H1299 cells transfected with a GPN3-overexpressing vector simultaneously expressing non-fused GFP were treated with 10 ng/ml EGF for 5 min and subjected to immunofluorescent staining. The arrow indicates GPN3-overexpressing cells, while asterisk indicates control cells. The dashed lines indicated cell outlines. **G** Overexpression of GPN3 leads to accumulation of EGFR on the cell membrane. The intensities of surface EGFR were normalized to corresponding total EGFR levels. **H** Upregulation of GPN3 leads to reduced endocytic EGFR. The intensities of endocytic EGFR were normalized to corresponding total EGFR levels.

Consideration that clathrin-mediated endocytosis is a primary pathway for ligand-induced EGFR endocytic trafficking [18], led us to hypothesize that GPN3 regulates EGF-induced EGFR endocytosis. To validate this hypothesis, we examined the impact of GPN3 overexpression on the co-localization of EGFR and CLTA following EGF stimulation. As shown in Fig. 2A, control cells exhibited co-localization of EGFR and CLTA upon EGF induction, but this phenotype was inhibited by GPN3 overexpression, suggesting that upregulation of GPN3 restricts the localization of EGFR within clathrin-coated vesicles and subsequent endocytic processes. Subsequently, we investigated the interaction between GPN3 and EGFR. Our findings demonstrated an interaction between EGFR and GPN3 (Fig. 2B, C), which was enhanced upon EGF stimulation (Fig. 2D). Additionally, an immunoprecipitation assay using endogenous proteins confirmed the interaction between GPN3 and EGFR (Fig. 2E). Furthermore, elevated levels of GPN3 suppressed the localization of EGFR in early endosomes following EGF stimulation (Fig. 2F). Upregulation of GPN3 resulted in increased membrane localization of EGFR (Fig. 2G), while reducing its internalization through endocytosis (Fig. 2H). Collectively, our data indicate that GPN3 confines EGFR to the cell membrane and inhibits ligand-induced trafficking via clathrin-mediated endocytosis.

### GPN3 prolongs the activation of EGFR signaling depending on GTP abundance

**Fig. 3:**
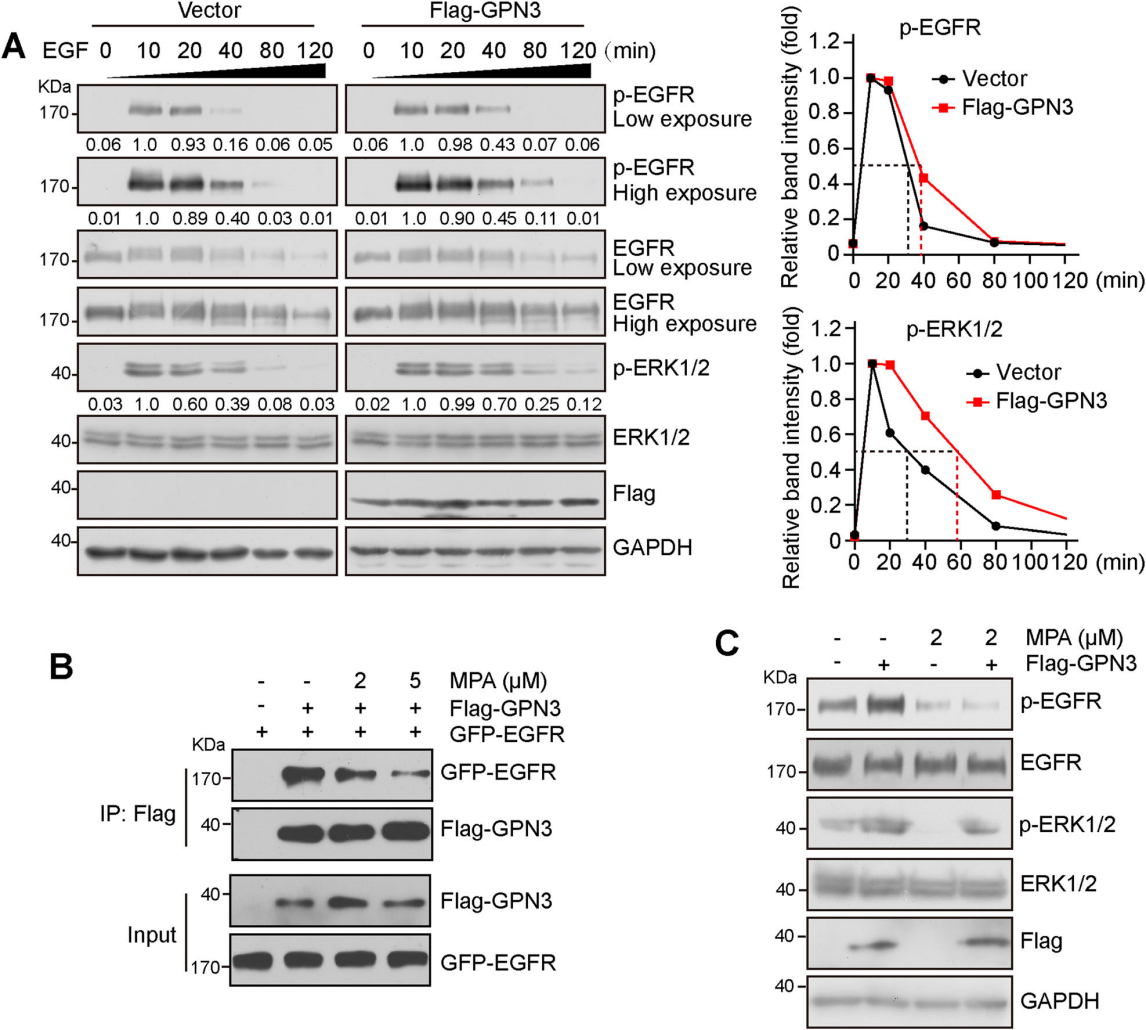
GPN3 prolongs the activation of EGFR signaling depending on cellular GTP abundance. **A** Overexpression of GPN3 prolongs the time course of phosphorylation of EGFR and downstream EKR1/2. 293T cells transfected with Flag-GPN3 or control vector were stimulated with EGF (10 ng/ml) for the indicated times. **B** MPA treatment attenuates the interaction between GPN3 and EGFR. 293T cells transfected with Flag-GPN3, GFP-EGFR or control vector were treated overnight with MPA and used to Co-IP assay. **C** MPA treatment inhibits the elevated phosphorylation levels of EGFR and ERK1/2 induced by GPN3 overexpression. 293T cells transfected with Flag-GPN3 or control vector were treated overnight with MPA, followed by stimulation with EGF (10 ng/ml) for 20 min prior to lysis, then the lysate was used for immunoblotting assay.

We further investigated the impact of GPN3 on EGFR signaling activation and observed that upregulation of GPN3 extended the duration of EGFR phosphorylation and downstream ERK1/2 activation (Fig. 3A). Most GTPases regulate cellular functions by cycling between an active, GTP-bound conformation and an inactive, GDP-bound conformation [22]. Hyperactivation of GTPases has been implicated in various human diseases, including cancers [5]. To deplete intracellular GTP levels, we employed mycophenolic acid (MPA), an inhibitor of inosine monophosphate dehydrogenase (IMPDH) [25], which disrupted the interaction between GPN3 and EGFR (Fig. 3B). Furthermore, MPA treatment reversed the enhanced activation of EGFR signaling mediated by GPN3 overexpression (Fig. 3C). Thus, our findings suggest that elevated expression of GPN3 promotes activation of EGFR signaling, which is dependent on the cellular GTP abundance, indicating that GPN3 mediated cellular functions may require an active GTP-bound state.

### Downregulation of GPN3 suppresses cell proliferation and migration in NSCLC cells

**Fig. 4:**
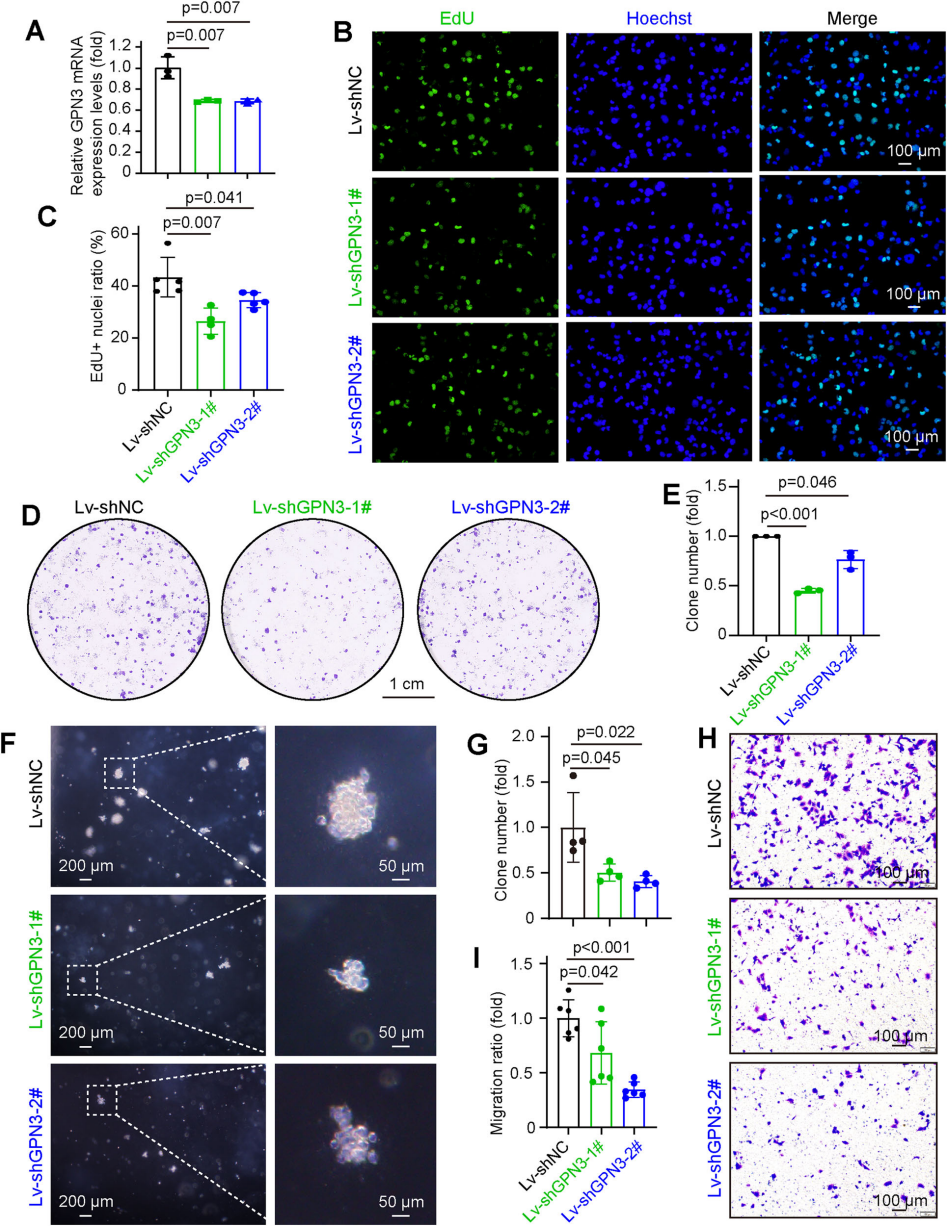
Downregulation of GPN3 suppresses cell proliferation and migration in NSCLC cells. **A** The mRNA level of GPN3 was detected in NCI-H1299 cells stably downregulating GPN3. NCI-H1299 cells were infected with lentivirus that downregulating GPN3 and used to real-time PCR assay. **B**, **C** Knockdown of GPN3 results in a significant reduction in the percentage of EdU-positive cells compared to control NCI-H1299 cells. **D** GPN3 knockdown inhibits colony formation ability in NCI-H1299 cells. **E** Quantification data for **D** is presented. **F** Depletion of GPN3 led to impaired colony formation ability in NSCLC cells under 3D culture conditions. **G** Colony numbers shown in **F** were quantified. **H** Downregulation of GPN3 significantly inhibits NSCLC cell migration. NCI-H1299 cells stably downregulated GPN3 were used to cell migration assay. **I** The migration ratio in **H** was quantified. **A**, **C**, **E**, **G**, **I** The data is presented as the mean ± SD, and *p* values were calculated by independent-samples *t-*test.

To investigate the impact of GPN3 on NSCLC malignancy, we established stable GPN3-downregulated NCI-H1299 and PC9 cell lines (Figs. 4A and S2A). The percentage of Edu incorporation was reduced in GPN3 downregulated NCI-H1299 cells compared to control cells (Fig. 4B, C). Colony formation assay revealed that downregulation of GPN3 inhibited the proliferative capacity of NCI-H1299 cells (Fig. 4D, E). Additionally, colony formation ability was also impaired in GPN3-knockdown NCI-H1299 cells under 3D culture conditions (Fig. 4F, G). Furthermore, cell migration ability was diminished in GPN3 downregulated cells compared to control NCI-H1299 cells (Fig. 4H, I). Similarly, downregulation of GPN3 resulted in suppressed cell growth (Fig. S2B), decreased percentage of EdU-positive cells (Fig. S2C, D), and reduced cell migration ability (Fig. S2E, F) when compared with control PC9 cells. Thus, these findings demonstrate the involvement of GPN3 in NSCLC progression by regulating malignant properties.

### GPN3 promotes the proliferation and migration of NSCLC cells both in vitro and in vivo

**Fig. 5:**
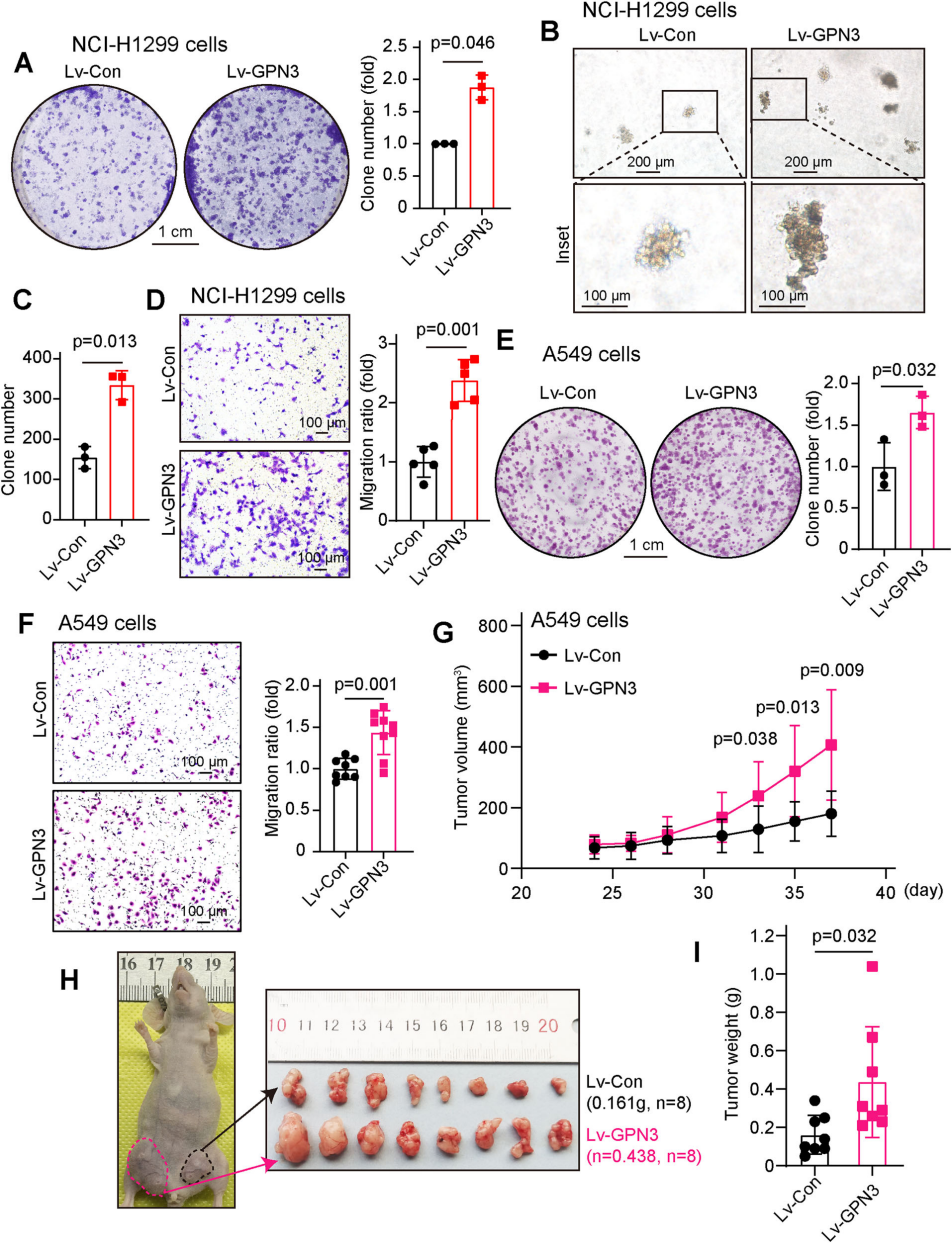
GPN3 accelerates NSCLC cell proliferation and migration in vitro and in vivo. **A** Overexpression of GPN3 enhances NSCLC cell proliferation. NCI-H1299 cells stably overexpressed GPN3 and control cells were used to colony formation assay. **B** Upregulation of GPN3 promotes NSCLC cell proliferation under 3D culture condition. NCI-H1299 cells infected with GPN3 overexpressing lentivirus and control cells were cultured in 0.3% agar. **C** The number of colonies from **B** was quantified. **D** GPN3 overexpression accelerates cell migration of the NSCLC cell line NCI-H1299. **E** Increased expression of GPN3 enhances NSCLC cell proliferation. A549 cells stably overexpressing GPN3 and control cells were subjected to colony formation assay. **F** Overexpression of GPN3 facilitates cell migration in NSCLC. **G**–**I** In vivo experiments demonstrate that GPN3 overexpression accelerates xenograft tumor growth in NSCLC models. Quantification of xenograft tumor volumes (**G**), representative images of xenograft tumors (**H**), and quantification of xenograft tumor weight (**I**) after subcutaneous transplantation into nude mice using A549 cells stably overexpressing GPN3 and control cells. **A**, **C**–**G**, **I** The data is shown as mean ± SD, and *p* values were determined by independent-samples *t-*test.

We further investigated whether GPN3 overexpression accelerates cell proliferation and migration of NSCLC cells, we stably overexpressed GPN3 in NSCLC cell lines NCI-H1299 and A549 using a lenti-virus system (Fig. S3A). Our results demonstrated that upregulation of GPN3 significantly increased the proliferation of NCI-H1299 cells (Fig. 5A–C) as well as facilitated their migration (Fig. 5D). Similarly, in NSCLC cell line A549, upregulation of GPN3 also accelerated cell proliferation (Fig. 5E) and migration (Fig. 5F). In the condition of EGF stimulation, GPN3 overexpression accelerated cell proliferation (Fig. S3B–D) and migration (Fig. S3E, F) of NCI-H1299 cells. Moreover, under EGF stimulation conditions, GPN3 overexpression further enhanced the activation of EGFR signaling pathway (Fig. S3A). These findings collectively highlight the crucial role played by GPN3 in promoting the proliferation and migration of NSCLC cells.

To investigate whether the NSCLC cellular phenotypes described above regulated by GPN3 could be reproduced in a more biologically significant system, xenograft tumor models were generated using an NSCLC cell line. A549 cells overexpressed GPN3 and control cells were subcutaneously injected into the flanks of nude mice, respectively. As shown in Fig. 5G–I, upregulation of GPN3 significantly enhanced the growth of NSCLC tumors. And, in GPN3-overexperssing xenograft tumors, the cell proliferation marker Ki67 and the key modulator of migration matrix metallopeptidase 9 (MMP9) were found to be overexpressed compared with those in the control group (Fig. S3G). These findings suggest that GPN3 plays a pivotal role in driving xenograft tumor growth in NSCLC.

### High level of GPN3 is associated with poor prognosis of NSCLC

**Fig. 6:**
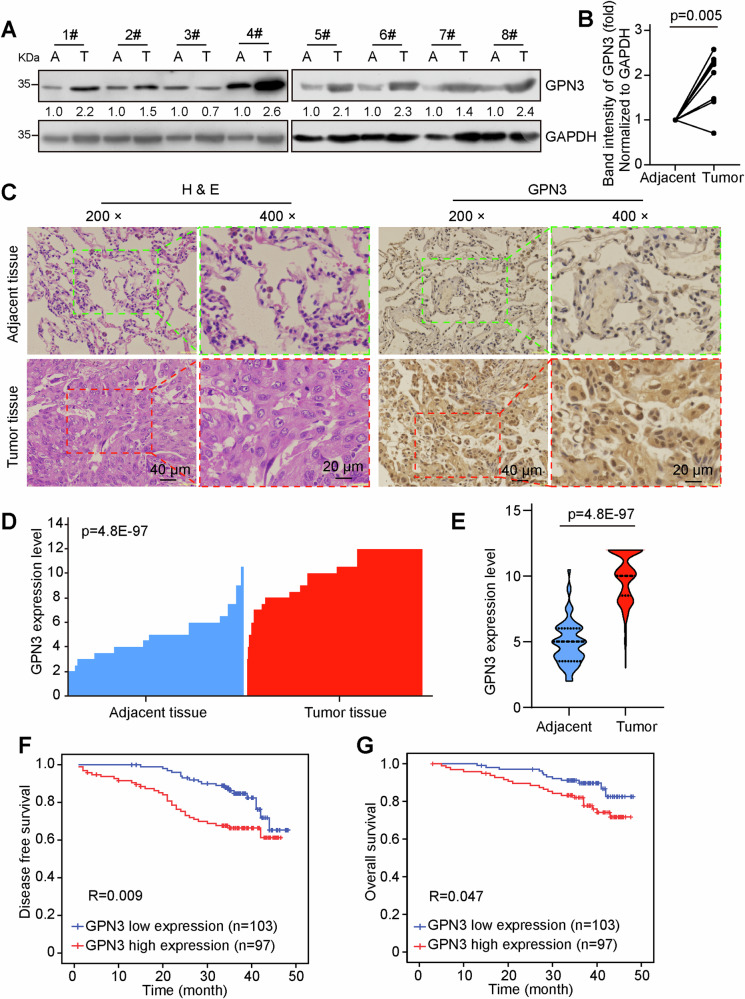
High level of GPN3 is associated with a poor prognosis of NSCLC. **A** NSCLC tissues exhibited elevated levels of GPN3 compared to their corresponding adjacent normal tissues. **B** The expression of GPN3 detected in **A** were quantified. The band intensities of GPN3 were normalized to the corresponding band intensity of GAPDH. The data is shown as mean ± SD, and *p* values were determined by independent-samples *t-*test. **C**–**E** GPN3 was higher expressed in NSCLC tissues compared with that in adjacent normal tissues. **C** Representative images are shown. **D** IHC scores of GPN3 expression in each NSCLC tissue sample and adjacent normal tissues sample were quantified. The p value was calculated by paired-samples *t-*test. **E** Violin plots showing the quantification of GPN3 expression in NSCLC tissues and adjacent normal tissues. The p value was calculated by paired-samples *t-*test. **F**, **G** High levels of GPN3 are associated with poor survival in NSCLC patients. Disease-free survival (**F**) and overall survival (**G**) of NSCLC patients were analyzed via univariate analysis. *P* values were quantified by the log-rank (Mantel-Cox) test.

To explore the potential involvement of GPN3 in the progression of NSCLC, we initially examined the expression level of GPN3 in two major NSCLC subtypes, namely LUAD and LUSC, utilizing the TCGA database through UALCAN analysis platform [26]. As shown in Fig. S4A, D, GPN3 exhibited upregulation in both LUAD and LUSC tissues compared to normal tissues, with higher stage NSCLC showing elevated expression levels of GPN3 (Fig. S4B, E). Western blot assay performed on 8 randomly selected paired NSCLC and adjacent normal tissues confirmed significantly higher expression of GPN3 in NSCLC tissues than their adjacent normal tissues (Fig. 6A, B). Additionally, IHC assay demonstrated an increased level of GPN3 expression in NSCLC tissues compared to adjacent normal tissues (Fig. 6C–E). High expression of GPN3 was strongly associated with distant metastasis and histologic grade of NSCLC, potentially correlated with differentiation as well (Table 1). Furthermore, high levels of GPN3 were linked to poor prognosis for patients with LUAD or LUSC through analysis conducted using GEPIA2 [27] and Kaplan-Meier Plotter [28], respectively (Fig. S4C, F). Consistently, we observed that the expression of GPN3 was related to shorter disease-free survival and overall survival rates among NSCLC patients (Table 2), where high expression levels resulted in reduced disease-free survival rates as well as overall survival rates (Fig. 6F, G). Collectively, these findings indicate that high expression levels of GPN3 contribute to the progression of NSCLC.

**Table 1:** Association of GPN3 expression level with different clinicopathologic characteristics in NSCLC patients.

	GPN3 expression					
	n	Low	n %	High	n %	p value
		count		count		
Gender						
Male	200	47	23.5%	52	26.0%	0.259
Female		56	28.0%	45	22.5%	
Age, years						
<60	200	53	26.5%	38	19.0%	0.081
≥60		50	25.0%	59	29.5%	
Smoking						
No	198	63	31.8%	52	26.3%	0.297
Yes		39	19.7%	44	22.2%	
Primary tumor size, cm						
≤3	200	68	34.0%	60	30.0%	0.54
>3		35	17.5%	37	18.5%	
Lymphatic metastasis						
No	200	70	35.0%	64	32.0%	0.766
Yes		33	16.5%	33	16.5%	
Distant metastasis						
No	200	85	42.5%	65	32.5%	0.011
Yes		18	9.0%	32	16.0%	
Intravascular tumor thrombus						
No	200	93	46.5%	90	45.0%	0.528
Yes		10	5.0%	7	3.5%	
Differentiation						
Well	200	39	19.5%	22	11.0%	0.064
Moderate		39	19.5%	44	22.0%	
Poor		25	12.5%	31	15.5%	
Histologic grade						
I	200	57	28.5%	43	21.5%	0.01
II		11	5.5%	12	6.0%	
III		18	9.0%	8	4.0%	
IV		17	8.5%	34	17.0%	
Radiotherapy						
No	194	90	46.4%	77	39.7%	0.104
Yes		10	5.2%	17	8.7%	
Chemotherapy						
No	198	61	30.8%	48	24.2%	0.166
Yes		41	20.7%	48	24.2%

**Table 2:** Univariate analysis of factors associated with recurrence and survival of NSCLC patients.

	Disease-free survival				Overall survival			
	HR	95% CI		p value	HR	95% CI		p value
		Lower	Upper			Lower	Upper	
Gender (male versus female)	1.528	0.881	2.651	0.131	1.438	0.736	2.811	0.288
Age (<60 versus ≥60 year)	0.710	0.411	1.225	0.218	1.958	0.959	3.998	0.065
Smocking (no versus yes)	1.326	0.765	2.298	0.315	1.644	0.847	3.191	0.142
Primary tumor size (≤3 versus >3 cm)	1.685	0.977	2.906	0.061	1.667	0.857	3.240	0.132
Lymphatic metastasis (no versus yes)	2.965	1.716	5.122	<0.0001	2.697	1.386	5.249	0.004
Distant metastasis (no versus yes)	43.865	18.520	103.893	<0.0001	6.083	3.025	12.233	<0.0001
Intravascular tumor thrombus (no versus yes)	2.847	1.336	6.069	0.007	1.550	0.547	4.394	0.410
Differentiation (well, moderate, and poor)	1.802	1.246	2.607	0.002	2.233	1.386	3.596	0.001
Histologic Grade (I, II, III, and IV)	8.579	4.419	16.657	<0.0001	2.382	1.718	3.301	<0.0001
Radiotherapy (no versus yes)	6.183	3.499	10.925	<0.0001	2.372	1.091	5.157	0.029
Chemotherapy (no versus yes)	4.123	2.195	7.746	<0.0001	2.344	1.136	4.838	0.021
GPN3 expression (low versus high)	2.080	1.180	3.666	0.011	2.001	0.994	4.029	0.046

## Discussion

### GPN3 functions as an oncogene in NSCLC

GPN3 is evolutionarily conserved across eukaryotic cells, ranging from archaea to humans [11], indicating that it has fundamental cellular functions in human health and diseases, including cancers. However, to date, only two publications have demonstrated that constitutively activated GPN3 confers protection against chemoresistant small cell lung cancer cells [13] and promotes proliferation of breast cancer cells [14]. In this study, we observed higher expression levels of GPN3 in NSCLC tissues compared to their adjacent normal tissues (Fig. 6). Moreover, aberrantly elevated expression of GPN3 was associated with a poor prognosis for NSCLC patients (Fig. 6). And, Additionally, downregulation of GPN3 inhibited both proliferation and migration of NSCLC cells, while overexpression of GPN3 enhanced cell proliferation and migration in vitro and in vivo (Figs. 4 and 5). These findings collectively demonstrated that GPN3 acted as an oncogene in NSCLC, which may be a potential diagnostic and prognostic biomarker for NSCLC, suggesting it may be a therapeutic target for NSCLC treatment. Nevertheless, further investigations are warranted to elucidate the involvement of GPN3 in other tumor types as well as non-tumor diseases.

### GPN3 regulates vesicular transport and protein trafficking

Due to the critical cellular function of GTPases, extensive efforts have been made over the past three decades to elucidate their functions [29]. Based on evolutionary classification, the GTPases superclass has been divided into two major classes: TRAFAC (named after translation factors) and SIMIBI (named after signal recognition particle (SRP), MinD, and BioD) [4]. The TRAFAC class, which includes classic GTPases and the Ras superfamily, is involved in translation, intracellular transport, signaling transduction, and cell motility. The SIMIBI class consists of SRP GTPases and several ATPases family that participated in chromosome partitioning, protein localization, membrane transport, as well as kinase activity or related phosphate transferase activity. Evolutionarily, the GPN-loop GTPase subfamily belongs to the SIMIBI class, but its structure differs from other GTPases of SIMIBI class [8]. It has been reported that GPN3 mediates nucleocytoplasmic transport of RNA polymerase II [12], but whether it regulates membrane transport and protein localization needs to be further unearthed. In this study, our results demonstrate that upregulation of GPN3 inhibits CCPs invagination and EGFR localization within clathrin-related subcellular structures and early endosomes upon EGF stimulation (Figs. 1 and 2). Furthermore, overexpression GPN3 led to accumulation of EGFR on the cell surface membrane by inhibiting its endocytic transport process thereby prolonging EGFR signaling activation (Figs. 1–3). Taken together, these findings indicate that GPN3 participated in vesicular transport and protein trafficking.

Besides vesicle transport and protein trafficking, GPN3 may also participate in other molecular mechanisms based on the enriched terms of GPN3 potential interactors (Fig. 1A). For instance, as neutrophils are immune cells and serve as the first responders to inflammation [30], it remains unknown whether GPN3 influences the tumor microenvironment and mediates cancer metastasis by regulating neutrophil degranulation. Additionally, further investigation is required to verify if GPN3 modulates tumor angiogenesis through manipulation of VEGFA-VEGFR2 signaling, a crucial positive regulator of angiogenesis [31]. Moreover, confirmation is needed regarding whether GPN3 plays a role in tumor metabolic reprogramming by involvement in carbohydrate metabolism or monocarboxylic acid metabolic processes. Therefore, there are still intriguing functions of GPN3 that warrant exploration.

### Cellular GTP abundance is essential for the functionality of GPN3

Most GTPases regulate downstream effector proteins and elicit cellular responses by cycling between an active state bound to GTP and an inactive state bound to GDP [7, 32]. GPN-loop GTPases possess weak intrinsic GTPase activity, but nucleotide binding induces only minor structural changes [8]. The precise cellular function of GTP-bound needs further elucidation, Huang et al. demonstrated that a constitutively GTP-bound mutant of GPN3 protected chemoresistant small lung cancer cells [13]. In our study, we observed that depletion of GTP attenuated the interaction between GPN3 and EGFR and inhibited the activation of EGFR signaling mediated by overexpression of GPN3 (Fig. 3). Thus, similar to other GTPases, being in a bound state with GTP may be crucial for the functionality of GPN3.

In this study, GPN3 impedes clathrin-dependent endocytosis of EGFR, thereby disrupting the negative feedback regulation of EGFR signaling and leading to prolonged activation of EGFR signaling. Additionally, GPN3 promotes cell proliferation and migration in NSCLC, and its aberrant overexpression in NSCLC tissues is associated with a poor prognosis. Therefore, GPN3 may be a potential prognostic biomarker and therapeutic target for NSCLC.

## Materials and methods

### Antibodies and constructs

Antibodies were obtained from Cell Signaling Technology (phospho-EGFR(Tyr1068), #3777; phospho-ERK1/2 (Thr202/Tyr204), #4370; EGFR, #4267; ERK1/2, #4695), Proteintech (HA, 81290-1-RR; Flag, 66008-4-Ig; GST, 66001-2-Ig; GAPDH, 60004-1-Ig), BD Biosciences (EEA1, 610456), Sigma (GFP, G1544), and Sino Biological (GPN3, 201814-T40). And, secondary antibodies were purchased from Proteintech (HRP-conjugated anti-mouse secondary antibody, SA00001-1; HRP-conjugated anti-rabbit secondary antibody, SA00001-2) and Invitrogen (Alexa-Fluor-594 conjugated anti-rabbit secondary antibody, A-11012; Alexa-Fluor-594 conjugated anti-mouse secondary antibody, A11032; Alexa-Fluor-647 conjugated anti-rabbit secondary antibody, A31573).

To generate a human GPN3 overexpression plasmid, the full length of coding sequence of GPN3 was inserted into the region between the EcoRI and ClaI sites of pKH3-HA vector. To obtain plasmid that used to generate lentivirus for overexpression GPN3, the full length CDS sequence of human GPN3 was subcloned into the region between the NotI and NheI sites of pHAGE-CMV-MCS-IZsGreen vector. The full length CDS sequence of human GPN3 was also inserted into the region between the EcoRI and NotI sites of pGEX6P1 to obtain a plasmid expressing GST-GPN3. Human *EGFR* cDNA was purchased from Sino Biological (Beijing, China). Plasmids expressing HA-AP2B1, HA-GPN1, Flag-CLTA, and Flag-AP2S1 were obtained from Miaoling Biology (Wuhan, China). CLTA cDNA was subcloned into the region between the XhoI and EcoRI sites of pEGFP-C1. The sequences of all constructs were verified by sequencing.

### GST-pull down, coimmunoprecipitation (co-IP) and immunoblotting assay

The protein-protein interactions were determined using GST-pull down and co-IP, as described previously [33]. Briefly, for the GST-pull down assay, GST-GPN3 was purified using Anti-GST magnetic beads (HY-K0222, MedChemExpress, Shanghai, China), followed by incubating with lysates from 293T cells expressing HA-AP2B1, HA-GPN1, or Flag-CLTA. The bead-associated proteins were detected through immunoblotting assays.

Protein samples from either the bead-associated proteins or cell lysates containing a total of 100 μg protein were separated on 10% SDS-PAGE gels and transferred onto nitrocellulose membranes. Subsequently, the membranes were blocked with skim milk for 1 h and incubated overnight at 4 °C with primary antibodies. After incubation with HRP-conjugated secondary antibodies, the protein bands were visualized using medical X-ray film. The intensities of the blot bands were quantified using ImageJ software.

### Transmission electron microscope assay

CPPs invagination was detected by transmission electron microscope following a previously described protocol [34]. NCI-H1299 cells stably overexpressing GPN3 and control cells were collected and fixed at 4 °C for 4 h in an electron microscope fixative solution. After washing with PBS, the cell precipitation was pre-embedded in 1% agarose, postfixed with 1% OsO_4_ for 2 h at room temperature, dehydrate and embedded in resin. The embedded samples were then polymerized in a 60°C oven for more than 48 h before being sectioned into slices of thickness ranging from 60 to 80 nm. The sections were subjected to 2% uranium acetate saturated alcohol solution avoid light staining and 2.6% lead citrate avoid CO_2_ staining. Finally, the images were captured using a transmission electron microscopy (HT7800, HITACHI, Tokyo, Japan).

### Cell surface biotinylation assay and internalization assay

EGFR levels on the cell surface were measured using a cell surface biotinylation assay as described previously [35, 36]. NCI-H1299 cells stably overexpressed GPN3 and the control cells were washed and incubated with sulfo-NHS-SS-Biotin (0.5 mg/ml, 21331, Thermo Scientific, Cleveland, OH, USA) on ice for 40 min. Subsequently, excess sulfo-NHS-SS-biotin was removed by washing the cells and then blocked with NH_4_Cl (50 mM). Finally, the cells were lysed and the biotinylated proteins were captured at 4 °C overnight using Streptavidin Dynabeads (Invitrogen), followed by immunoblotting assay to determine EGFR expression on the cell surface membrane.

Endocytic EGFR was determined as previously described [36]. NCI-H1299 cells stably overexpressed GPN3 and the control cells on 10 cm dishes were surface biotinylated on ice with Sulfo-NHS-SS-biotin (0.5 mg/mL). The cells were subsequently washed PBS and any excess biotin was quenched using glycine (100 mM). Then, the cells were treated with 10 ng/ml EGF for different time periods to allow internalization of surface proteins. After washing with ice-cold PBS, surface-bound biotins were cleaved by incubation with reducing agent sodium 2-mercaptoethanesulfonate (MesNa) (10 mM, dissolved in 50 mM Tris (pH = 8.6), 150 mM NaCl, 1 mM EDTA and 0.2% BSA) at 4 °C for 20 min. Subsequently, an additional 10% volume of MesNa (100 mM) in same buffer was added and incubated for a further 20 min, and another 12.5% volume of MesNa (100 mM) was added. Finally, the cells were incubated with 16% volume of iodoacetamide (500 mM) to quench reducing agent, washed and lysed. The biotinylated proteins were captured at 4 °C overnight using Streptavidin Dynabeads (Invitrogen), followed by immunoblotting assay to detect biotinylated EGFR.

### Immunofluorescence assay

To investigate the regulatory effects of GPN3 overexpression on the co-localization of EGFR and CLTA, NCI-H1299 cells stably overexpressed GPN3 and the control cells transfected with GFP-CLTA were treated with EGF (10 ng/ml) for 5 min. To assess the impact of GPN3 overexpression on localization of EGFR in the early endosome, NCI-H1299 cells transfected with GPN3 (co-expressing non-fused GFP) were treated EGF (10 ng/ml) for 5 min.

For immunofluorescent staining, the cells cultured on coverslips were fixed with 4% PFA for 10 min and permeabilized using 0.5% TritonX-100 for 3 min. Then, the cells were block with 5% BSA and incubated overnight at 4°C with primary antibodies. After washing three times with PBS, slides were incubated at 4 °C for 4 h with Alexa Fluor-conjugated anti-rabbit/mouse secondary antibodies (488, 594 or 647 nm). Representative images were captured using a confocal microscope (Stellaris 5, Leica, Deerfield, IL, USA).

### Cell culture, cell transfection and lenti-virus infection

The human NSCLC cell lines A549, NCI-H1299 and PC9 used in this study were originated from the cell bank of the Chinese Academy of Sciences. The human embryonic kidney HEK-293T cell line was obtained from the American Type Culture Collection (ATCC, Manassas, VA). These cells were cultured at 37 °C in a humidified incubator with 5% CO_2_ using the recommended medium containing 10% fetal bovine serum. All cell lines underwent STR analysis to confirm their identity.

Transient transfection of HEK-293T was performed using PEI (408727, Sigma-Aldrich, St. Louis, MO), following manufacturer’s instructions. Lentiviral expression system was employed to establish stable overexpression or knockdown models of GPN3 in NSCLC cells. Lentivirus for GPN3 overexpression or knockdown were generated and packed, and lentivirus infection was carried out as previous publication [37]. The shRNA sequences targeting GPN3 were as follows: shGPN3-1#: 5’-TTAGCCTTCGCGCGACGCCCA-3’, and shGPN3-2#: 5’-GAACCAAAGGAACGTGAAG-3’. A scrambled sequence 5′-TTCTCCGAACGTGTCACGT-3′ was used as a negative control (shNC). Constructed NSCLC cells with stable overexpression or downregulation of GPN3 were confirmed by immunoblotting or qPCR assay. The qPCR primers for GPN3 and β-ACTIN are listed in Supplementary Table S1.

### Cell proliferation and migration assay

To determine the effects of GPN3 on NSCLC cell proliferation, CCK8, EdU incorporation, plate colony formation, and soft agar colony formation assays were carried out. For the CCK8 assay, the optical density (OD) at 450 nm was measured by a microplate reader (Varioskan LUX, Thermo Scientific, USA). EdU is a thymidine analog that is incorporated into the dividing cells’s DNA. Therefore, the percentage of EdU-positive (EdU + ) cells represents their proliferative ability. The EdU incorporation assay was conducted using a Cell-Light EdU Apollo567 in vitro kit (C10310-1, Ribo Biotechnology, Guangzhou, China) according to the manufacturer’s instructions. For the plate colony formation assay, 2000 cells were seeded into each well of a 6-well plate. After culturing with complete medium for 6 days, the cells were stained with crystal violet. To perform the soft agar colony formation assay, a 0.5% agar (A7002, Sigma-Aldrich, St. Louis, MO, USA) solution (dissolved in a complete medium) was added to a 12-well plate, 3000 cells were then resuspended with 0.3% agar solution (dissolved in a complete medium) and added to the top of the solidified 0.5% agar. After 10 days of culture, colonies with diameters greater than 50 μm were count and photographed with a microscope.

Cell migration assays were performed with Transwell plates with 8 μm pores (353097, Falcon, Durham, NC). Briefly, 30 000 cells resuspended in 200 μl of 1% FBS medium were added to the upper chambers while the bottom chambers were filled with 600 μl of complete medium containing 10% FBS. After a culture period of 24 h, the PET membrane of Transwells was fixed and stained with crystal violet. Subsequently, photographs of the cells on the lower surface of the membrane were captured using a microscope.

### Ethical statement and patients’ clinical specimens

The experimental protocol of subcutaneous xenograft tumor model was approved by and the assays were performed in accordance with the guidelines of the Institutional Animal Care and Use Committee of The First Affiliated Hospital of Nanchang University [No. CDYFY-IACUC-202311QR008].

Surgically resected human NSCLC tissues and adjacent tissues along with adjacent tissues from chemotherapy or radiotherapy-naive patients were collected at The First Affiliated Hospital of Nanchang University. Informed consent was obtained from all participants. The protocol for this work was approved by and the assays were conducted in accordance with the guidelines of the Medical Ethics Review Committee of The First Affiliated Hospital of Nanchang University [No. (2024)CDYFYYLK(02-016)].

### Subcutaneous xenograft tumor model

Subcutaneous xenograft tumor models were established in female BALB/c athymic (nu/nu) mice aged between 4 and 5 weeks old. The A549 cells were infected with lentivirus to generate GPN3-overexpressing (Lv-GPN3) and control (Lv-Con) cell lines. A suspension containing 1 × 10^7^ cells in a volume of 200 μl was subcutaneously injected into nude mice flanks for tumor formation assessment. Tumor volumes were measured using calipers and quantified according to the formula: length(mm) × width^2^(mm^2^)/2.

### Immunohistochemistry (IHC) assay

Immunohistochemical staining was performed according to previously published methods [33, 35], and two clinical pathology researchers were invited to assess the intensity and extent of staining in each sample. The final immunostaining score (GPN3 level) was calculated by multiplying the mean of intensity by the mean of extent. A primary antibody against GPN3 (1:50) was used to the IHC assay.

### Statistical analysis

The data were presented as mean ± SD from experiments conducted more than three times. Statistical analyses were performed using SPSS v.13.0 software (SPSS Inc., Chicago, IL). Independent samples t-tests were used to compare differences between two groups, while paired samples t-tests were used for paired comparisons. A p-value < 0.05 was considered statistically significant.

## Supplementary information

Supplemental Material

Original Data

## Data Availability

All data generated or analyzed during this study have been included in this article. Supplementary figures and tables, and full uncropped western blots were shown in the Supplementary Material.
