# Supplementary material for: GTPase GPN3 facilitates cell proliferation and migration in non-small cell lung cancer by impeding clathrin-mediated endocytosis of EGFR
Source: Cell Death Discov. 2025 Feb 1;11:38. doi: 10.1038/s41420-025-02317-y (PMC11787391; doi:10.1038/s41420-025-02317-y)
Supplement: Supplementary file 1 — Supplemental Material [file 41420_2025_2317_MOESM1_ESM.pdf]

Supplemental Material

GTPase GPN3 facilitates cell proliferation and migration in non-small cell lung cancer by impeding clathrin-mediated endocytosis of EGFR

Linlin Xu[1,2,#], Jiankun Guo[1,3,#], Xinsheng Xie[1,#], Hailong Wang[1], Alan Jiang[1], Changhua Huang[1,3], Hua Yang[1,3], Shiwen Luo[3,*], Limin Chen[1,*]

[1] Medical Innovation Center, The First Affiliated Hospital, Jiangxi Medical College, Nanchang University, Nanchang, Jiangxi 330006, China.

[2] Department of Pathology, The First Affiliated Hospital, Jiangxi Medical College, Nanchang University, Nanchang, Jiangxi 330006, China.

[3] Center for Experimental Medicine, The First Affiliated Hospital, Jiangxi Medical College, Nanchang University, Nanchang, Jiangxi 330006, China.

[#] These authors contributed equally to this work.

[*] **Corresponding author:** Limin Chen, PhD; Shiwen Luo, PhD

E-mail: liminchen@ncu.edu.cn (L.M. Chen), shiwenluo@ncu.edu.cn (S.W. Luo)

Mail Address: 17 Yongwai Street, Donghu District, Nanchang, Jiangxi 330006, China

Supplementary Table S1. Primers used in real time PCR assay.

Gene	Forward primer	Reverse primer
GPN3	CGGGAAGAGCACCTACTG	CATCCACCTCGATCAGTTCC
ACTIN	ACCTTCTACAATGAGCTGCG	CCTGGATAGCAACGTACATGG

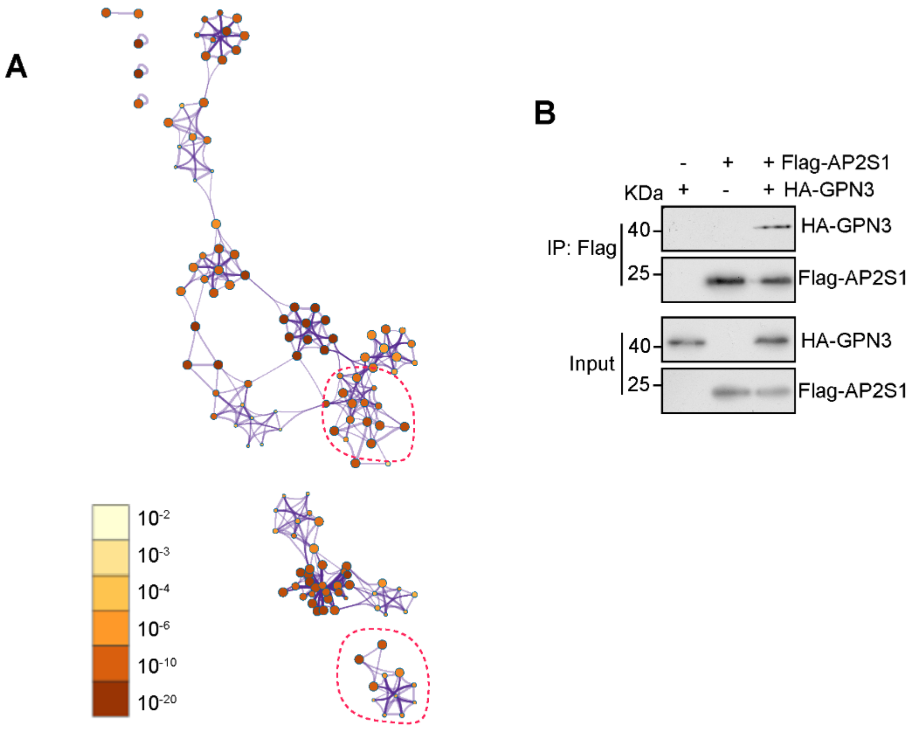

Figure S1. GPN3 regulates vesicle transport. (A) The top 20 enriched terms of GPN3-interacted hits identified by LC-MS/MS assay are presented, colored by p-value. (B) GPN3 interacts with AP2S1. 293T cells transfected with HA-GPN3, Flag-AP2S1, or control vector were lysed and used to immunoprecipitation using anti-Flag magnetic beads.

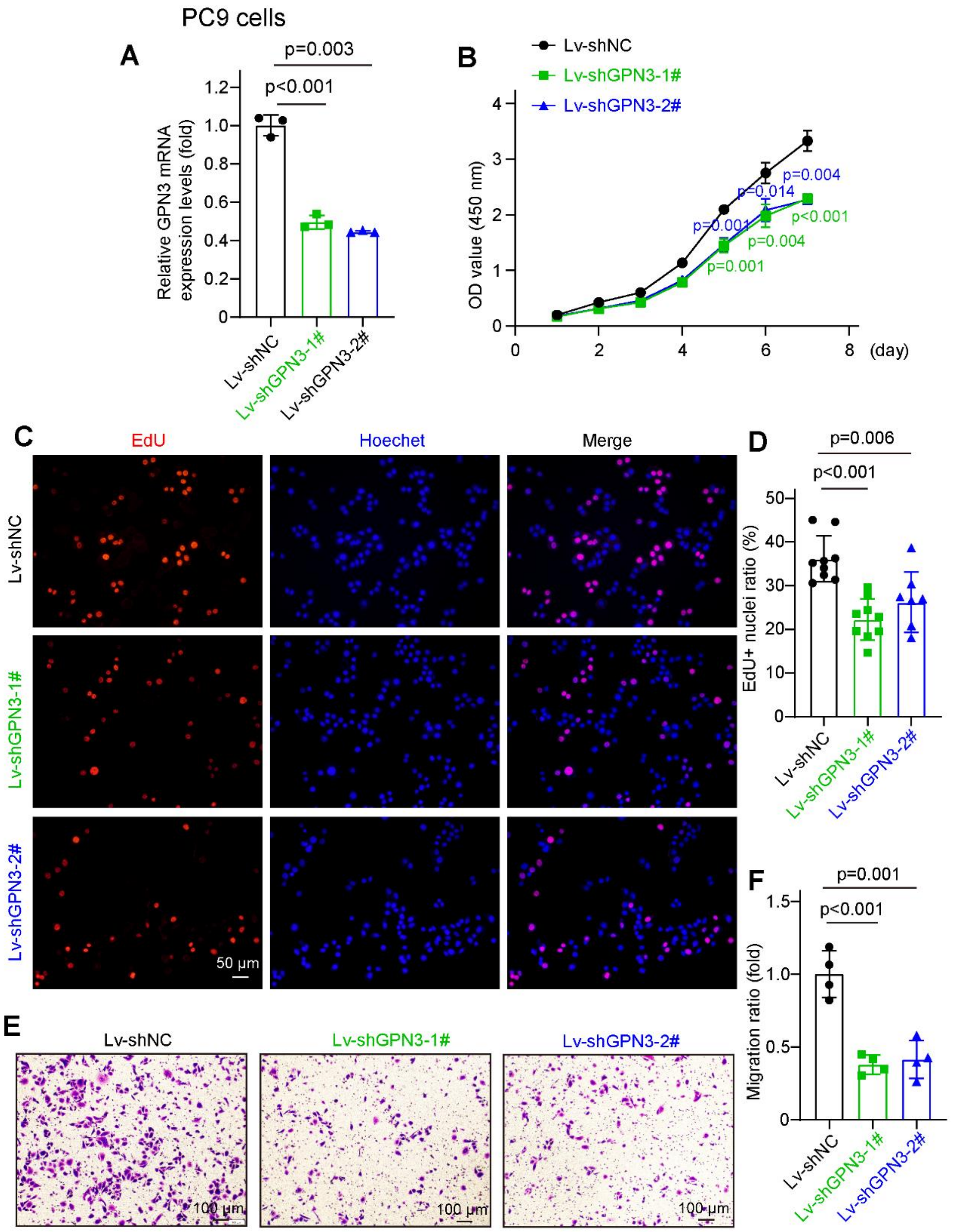

Figure S2. Downregulation of GPN3 restrains NSCLC cell proliferation and migration. (A) The mRNA level of GPN3 was detected in PC9 cells stably downregulating GPN3. PC9 cells were infected with lentivirus that downregulating GPN3 and subjected to qPCR assay. (B) GPN3 downregulation inhibits PC9 cell growth. (C-D) GPN3-downregulated cells exhibit decreased percentage of EdU-positive cells compared to control PC9 cells. (E-F)

Knockdown of GPN3 attenuates cell migration in PC9 cells. (A, B, D, F) The data is presented as mean±SD, and *p* values were calculated by independent-samples *t*-test.

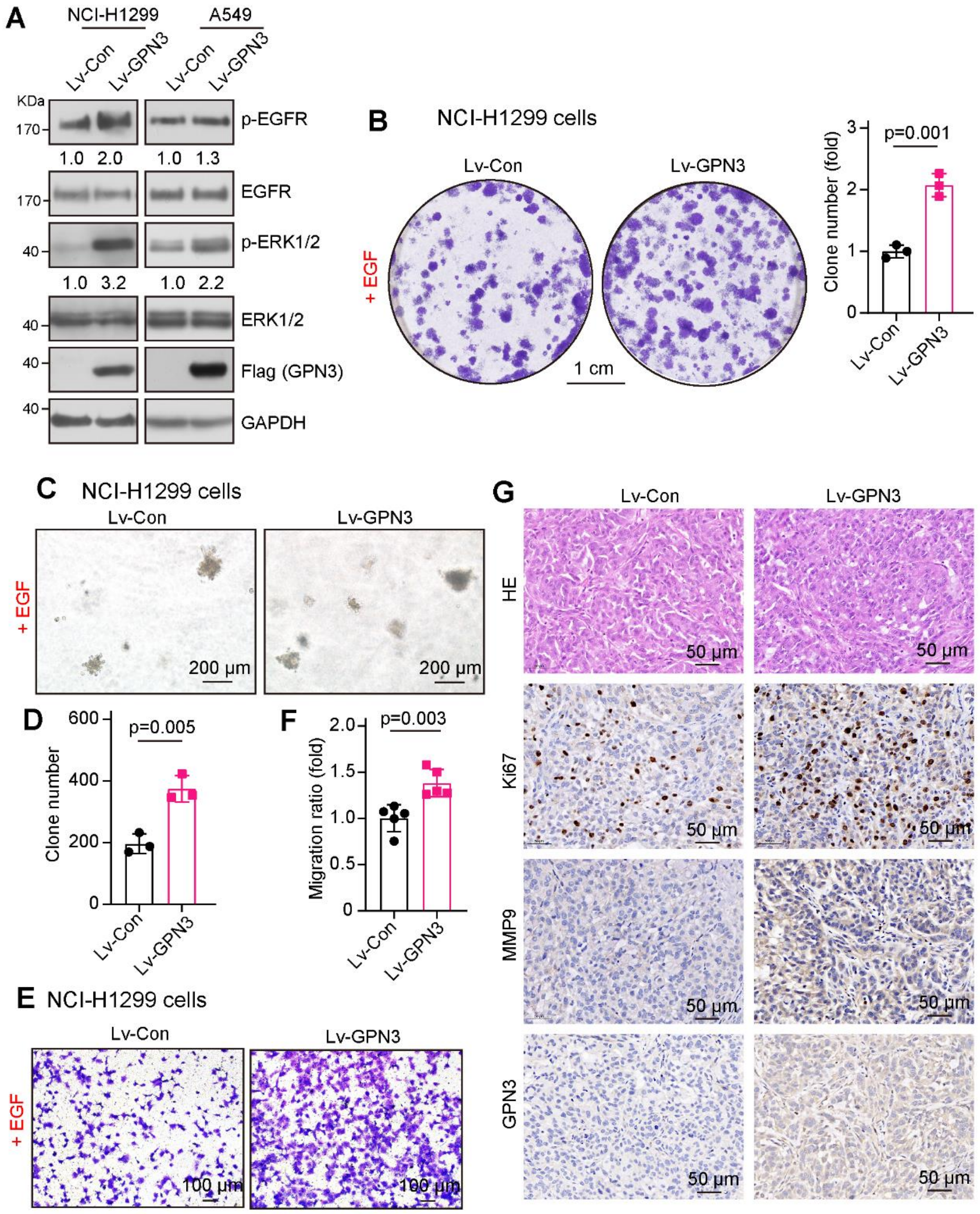

Figure S3. GPN3 overexpression promotes cell proliferation and migration of NSCLC cells upon EGF stimulation. (A) Upregulation of GPN3 augments EGFR signaling activation in NSCLC cell lines. NCI-H1299 and A549 cells stably overexpressing GPN3 were treated with EGF (10 ng/ml) for 20 min, followed by immunoblotting assay. (B) GPN3 overexpression promotes cell proliferation in NCI-H1299 cells upon EGF stimulation (10 ng/ml). (C-D) GPN3 overexpression accelerates cell proliferation in NCI-H1299 cells cultured in 3%

agar upon EGF stimulation (10 ng/ml). (E-F) GPN3-overexpressing cells exhibits enhanced migratory ability compared to control NCI-H1299 cells upon stimulation of EGF (10 ng/ml). (G) GPN3 promotes the proliferation and migration of NSCLC cells. The xenograft tumors were isolated, fixed, and used for detecting the expression of Ki67, MMP9, and GPN3 by IHC assay. (B, D, F) The data is presented as the mean±SD, and *p* values were determined by independent-samples *t*-test.

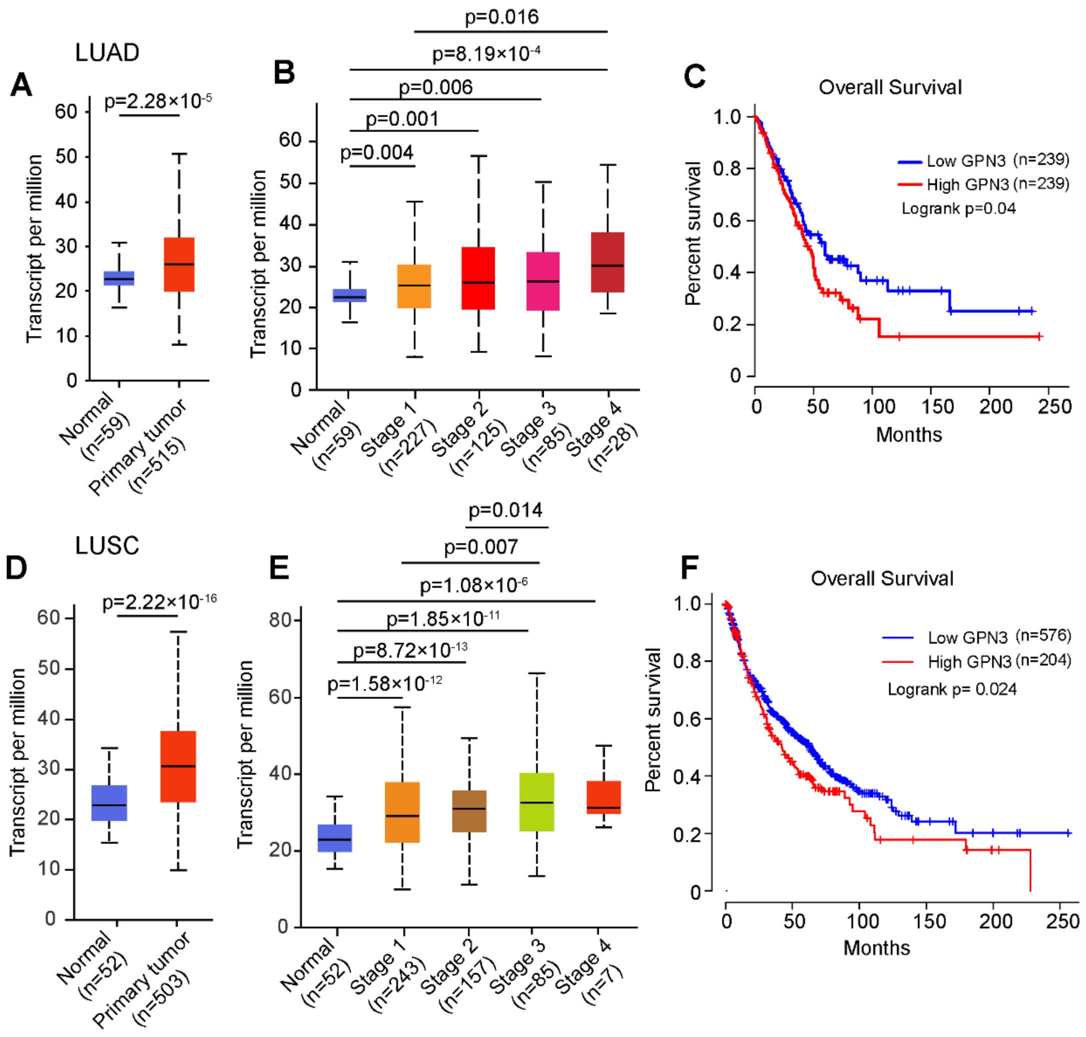

Figure S4. High expression of GPN3 is associated with a poor prognosis of NSCLC patients. (A) GPN3 was significantly highly expressed in LUAD tissues compared to normal tissues by analyzing TCGA database using UALCAN (https://ualcan.path.uab.edu/index.html). *P* values were determined by paired-samples *t*-test. (B) Higher stages of LUAD tissues are correlated with increased levels of GPN3 expression. (C) High expression of GPN3 was associated with a poor prognosis in LUAD patients. Kaplan Meier analysis was performed to analyze the overall survival incidence of patients with LUAD via GEPIA 2 (http://gepia2.cancer-pku.cn/). The *p* value was calculated by the log-rank (Mantel-Cox) test. (D) GPN3 was significantly highly expressed in LUSC tissues compared to normal tissues by analyzing TCGA database using UALCAN. *P* values were determined by paired-samples *t*-test. (E) GPN3

expression is positive related with tumor stage of LUSC. (F) High expression of GPN3 is associated with a poor prognosis in LUAD patients. Kaplan Meier analysis was used to analyze the overall survival incidence of patients with LUSC via Kaplan-Meier Plotter (https://kmplot.com/analysis/index.php?p=background). The *p* value was determined by the log-rank (Mantel-Cox) test.
