# Supplementary figures and images for: GTPase GPN3 facilitates cell proliferation and migration in non-small cell lung cancer by impeding clathrin-mediated endocytosis of EGFR
Source: Cell Death Discov. 2025 Feb 1;11:38. doi: 10.1038/s41420-025-02317-y (PMC11787391; doi:10.1038/s41420-025-02317-y)

**Full and uncropped Western blots**


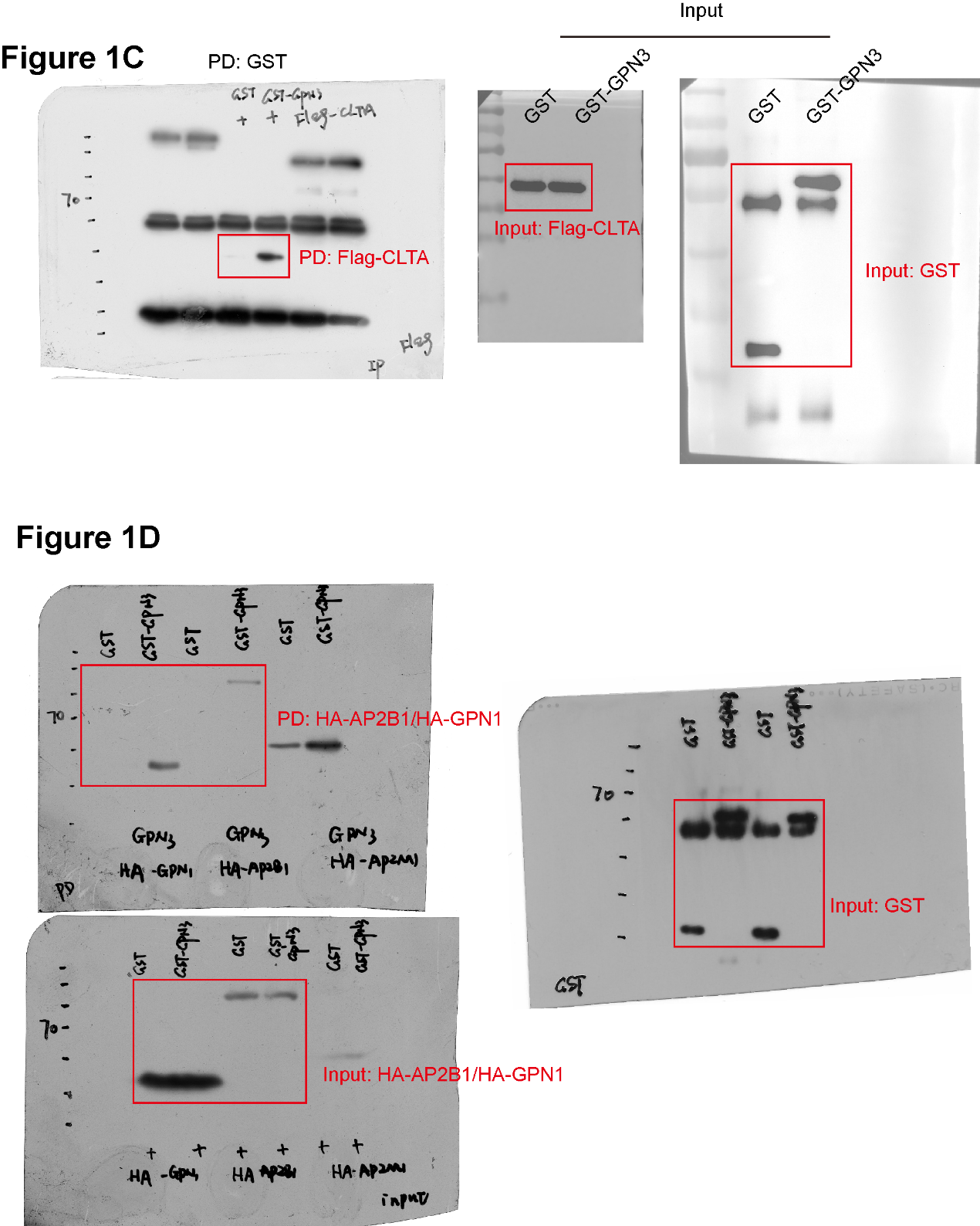


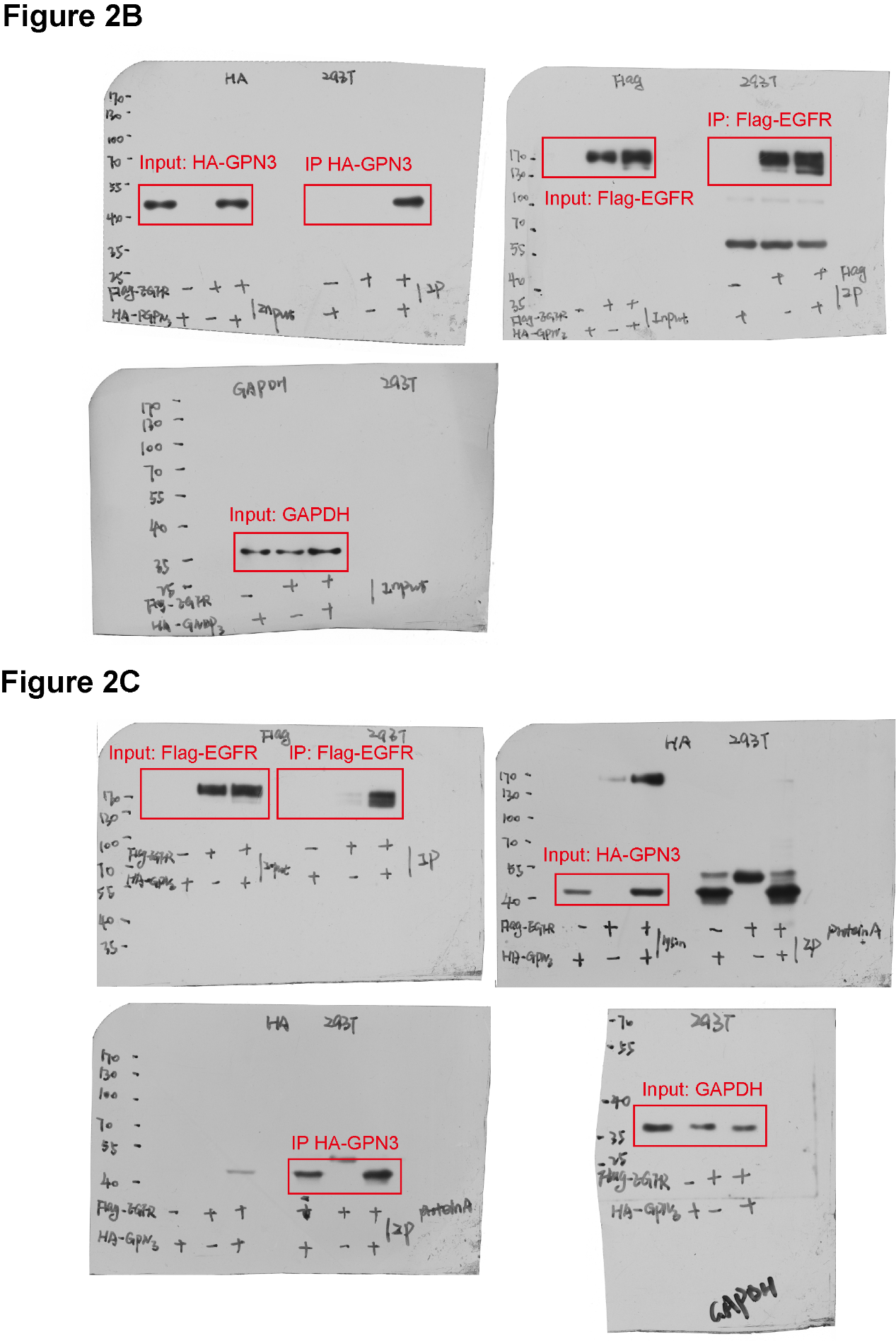


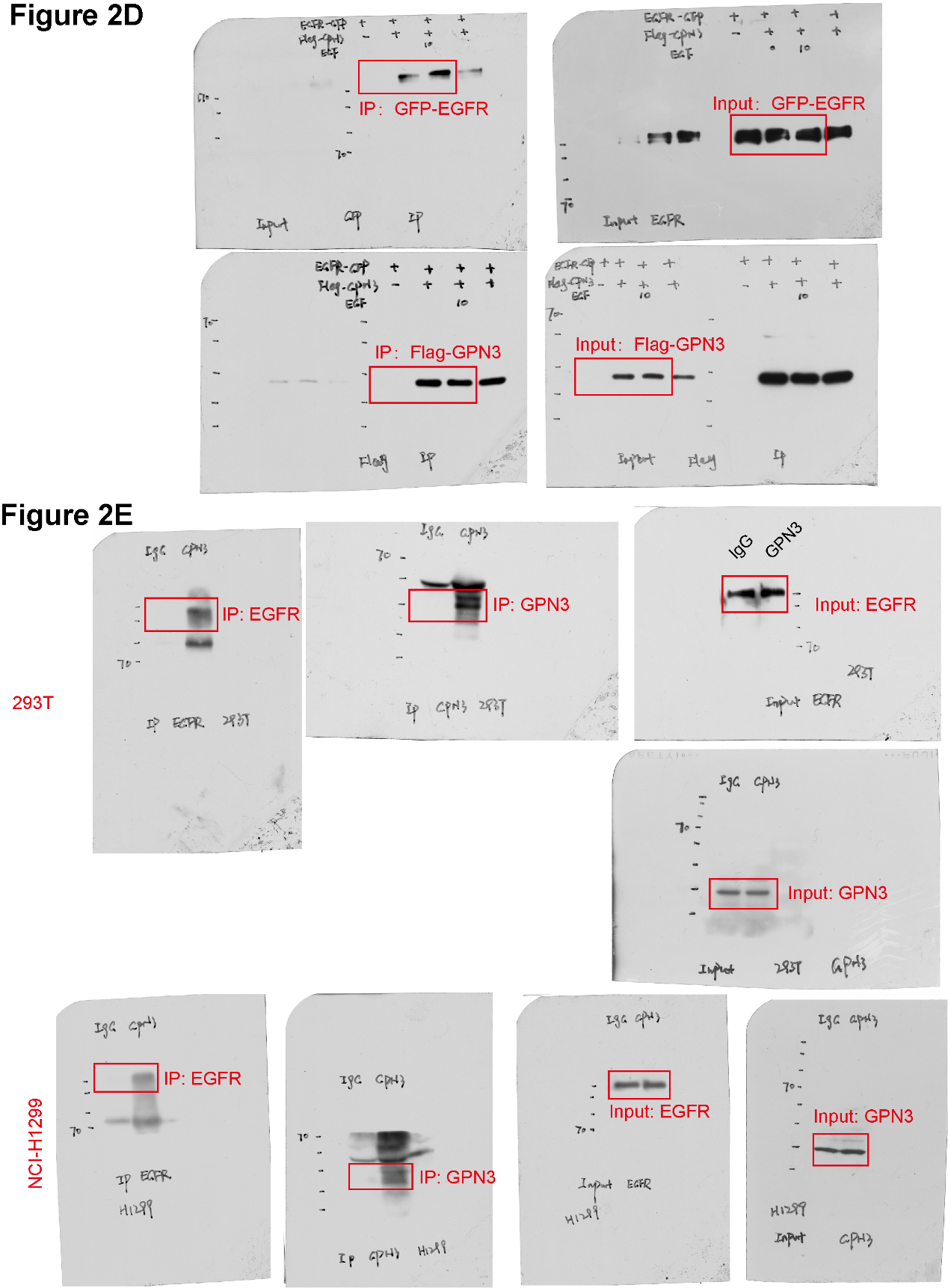


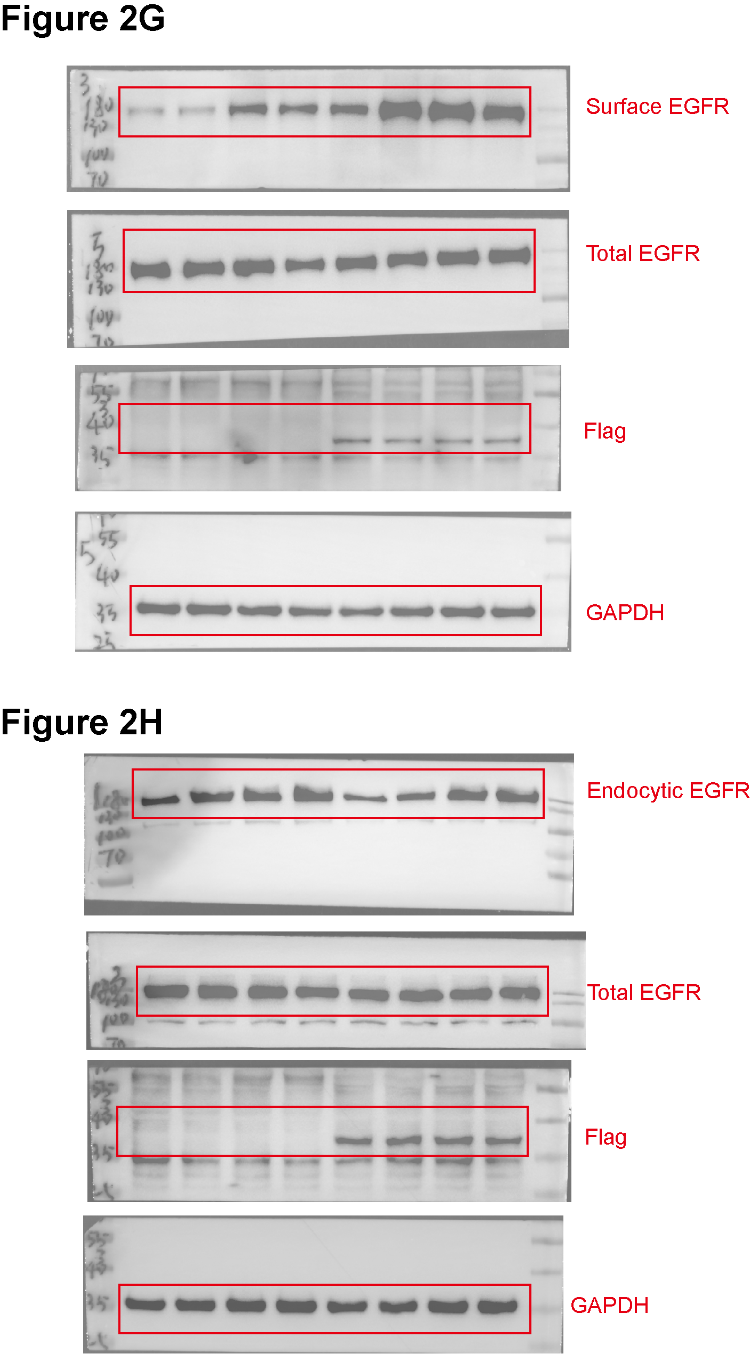


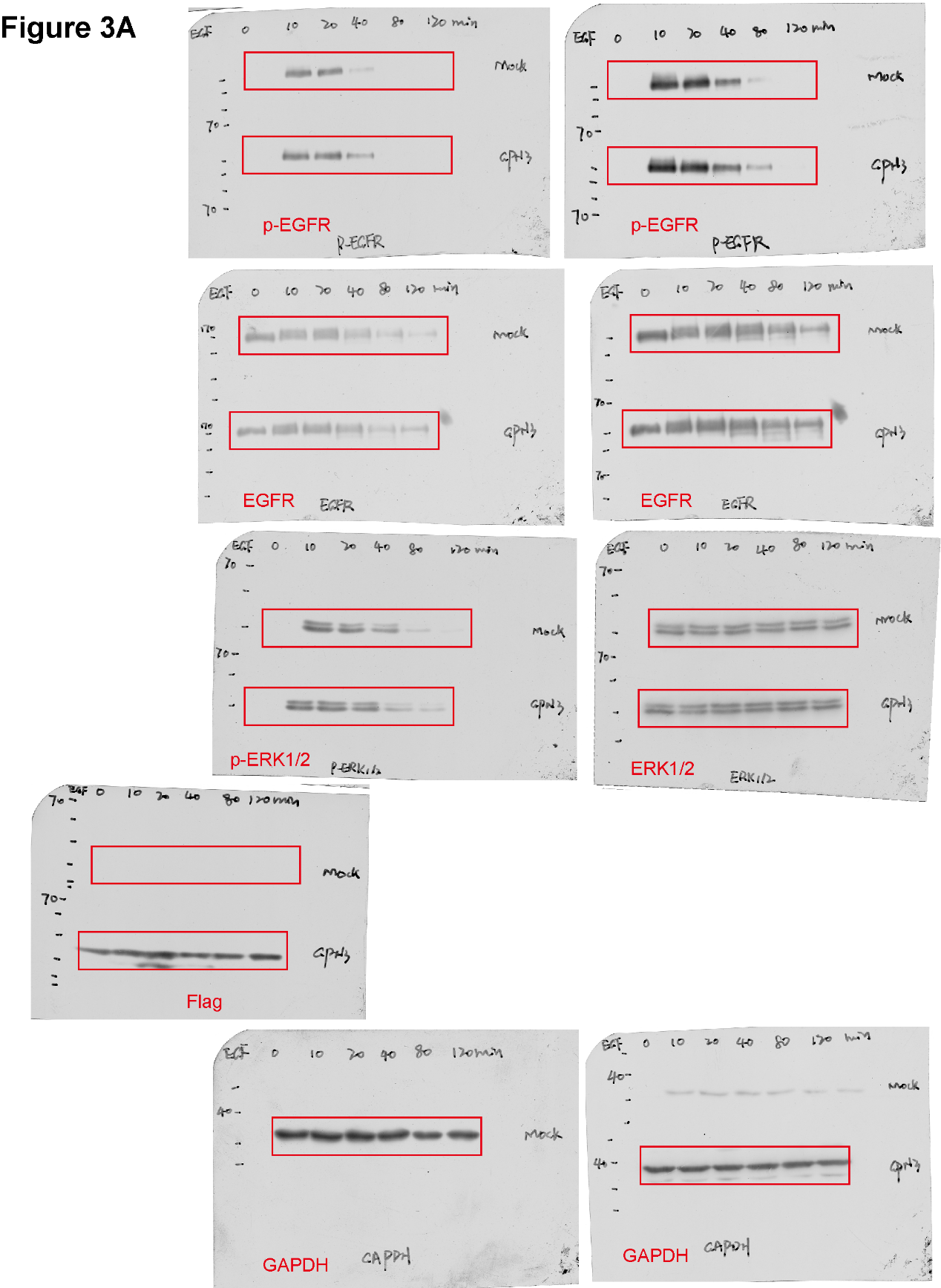


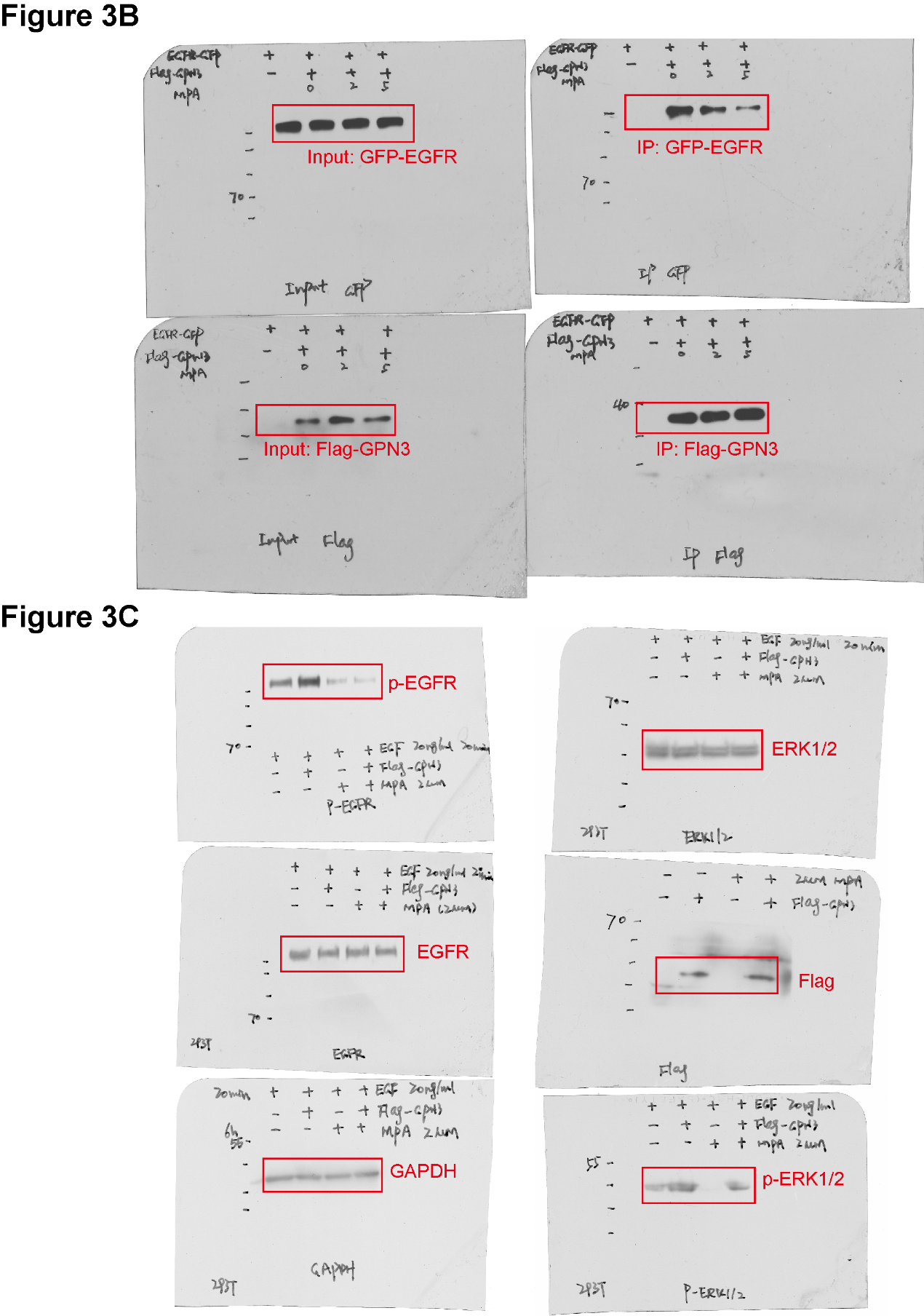


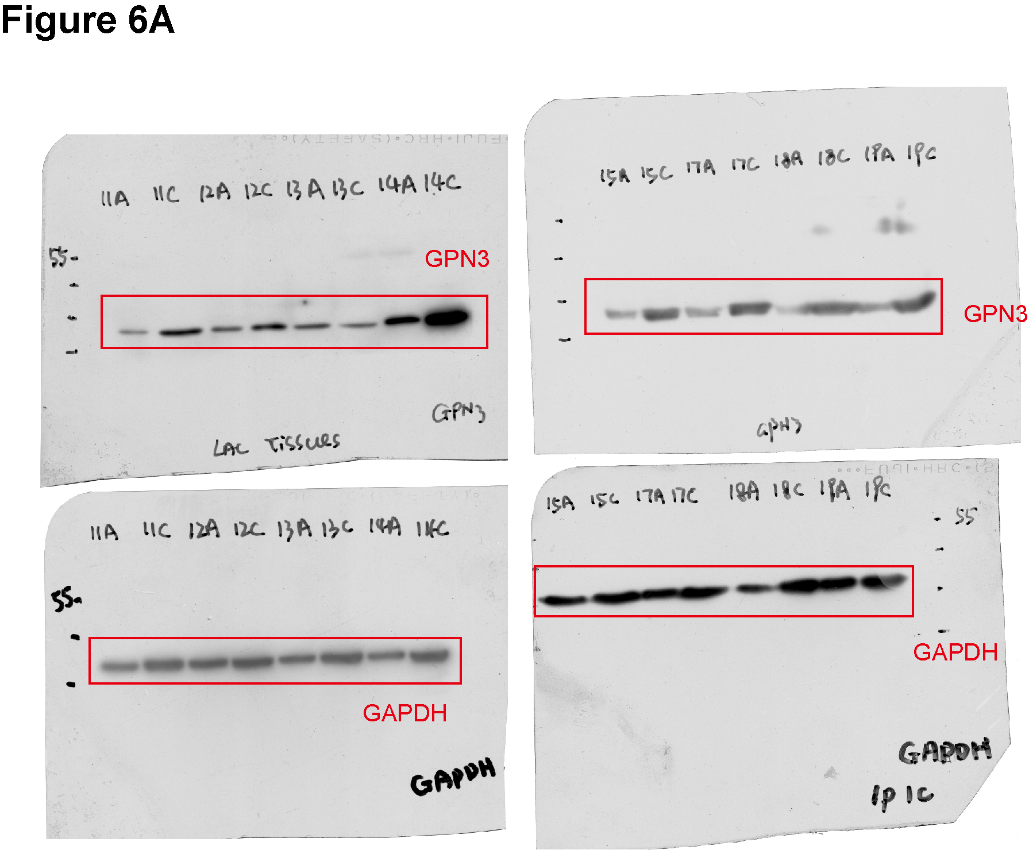

Supplement: Supplementary file 2 — Original Data [file 41420_2025_2317_MOESM2_ESM.docx]
